# Safety, pharmacokinetics and antiviral activity of AHB‑137 in healthy volunteers and chronic hepatitis B patients: a phase 1a/1b study

**DOI:** 10.1007/s12072-026-11102-7

**Published:** 2026-05-23

**Authors:** Wen Wang, Jiajia Mai, Jinlin Hou, Zhongyuan Xu, Xian Yu, Hong Ren, Xieer Liang, Yi Yang, Zhihong Liu, Hong Zhang, Shan Zhong, Yilei Wen, Tingting Lu, Haixia Cao, Xiao Qiu, Lidan Wang, Di Zhao, Miao Wang, Chengyong Yang, Guofeng Cheng, Junqi Niu, Yanhua Ding

**Affiliations:** 1 Phase I Clinical Trial Center, The First Hospital of Jilin University, No. 1 Xinmin Street, Changchun, Jilin China; 2 Nanfang Hospital, Southern Medical University, Guangzhou, China; 3 The Second Affiliated Hospital, Chongqing Medical University, Chongqing, China; 4 Ausper Biopharma Co. Ltd., Hangzhou, China; 5 AusperBio Therapeutics Inc., Foster City, CA USA; 6 Department of Hepatology, Center of Infectious Diseases and Pathogen Biology, The First Hospital of Jilin University, Changchun, China

**Keywords:** Antisense oligonucleotide, AHB-137, Chronic hepatitis B, Functional cure, Hepatitis B surface antigen, HBsAg reduction, HBsAg loss, Pharmacokinetics, Phase 1, Tolerability

## Abstract

**Background/purpose:**

AHB-137 is a novel antisense oligonucleotide targeting a conserved 3’-terminal region of HBV mRNA. This phase 1a/1b study evaluated the safety, pharmacokinetics (PK), and antiviral activity of AHB-137 in Chinese healthy volunteers (HVs) and chronic hepatitis B (CHB) patients.

**Methods:**

In Phase 1a, 52 HVs received single ascending doses (75–450 mg) or multiple ascending doses (MAD; 150 or 300 mg) of AHB-137 or placebo. In Phase 1b, 20 HBeAg-negative, nucleos(t)ide analogue (NA)-suppressed CHB patients with baseline HBsAg 100–1000 IU/mL received MAD AHB-137 (150 or 300 mg) or placebo (8:2), and 2 patients with baseline HBsAg 1000–3000 IU/mL received open-label AHB-137 300 mg. MAD comprised 6 subcutaneous doses over 4 weeks including loading doses on Days 4 and 11, with safety, PK and virologic follow-up assessments.

**Results:**

No serious adverse events, deaths or discontinuations occurred. Most treatment-related adverse events were Grade 1–2, including injection site reactions, pyrexia, and asymptomatic lab abnormalities. Transaminase elevations were self-limiting; one Grade 3 ALT elevation in the 300 mg CHB cohort coincided with rapid HBsAg loss. PK showed rapid absorption (T_max_ 3–5 h), dose-proportionality, and a terminal half-life of 128–195 h without meaningful accumulation. In CHB patients, 4 weeks of AHB-137 induced dose-dependent HBsAg reductions (mean maximum − 1.1 vs. − 2.1 log_10_ IU/mL at 150 vs. 300 mg), 50.0% of the 300 mg cohort achieved ≥ 2 log_10_ IU/mL reduction. Preliminary HBsAg loss was observed in a small subset of patients during follow-up.

**Conclusion:**

AHB-137 demonstrated favorable safety, predictable PK, and dose-dependent HBsAg reductions, including HBsAg loss, supporting its further development for CHB functional cure.

**Registration number:**

ClinicalTrials.gov. number: NCT06115993. Chinadrugtrials.org.cn. number: CTR20232098.

**Graphical abstract:**

**Figure:**
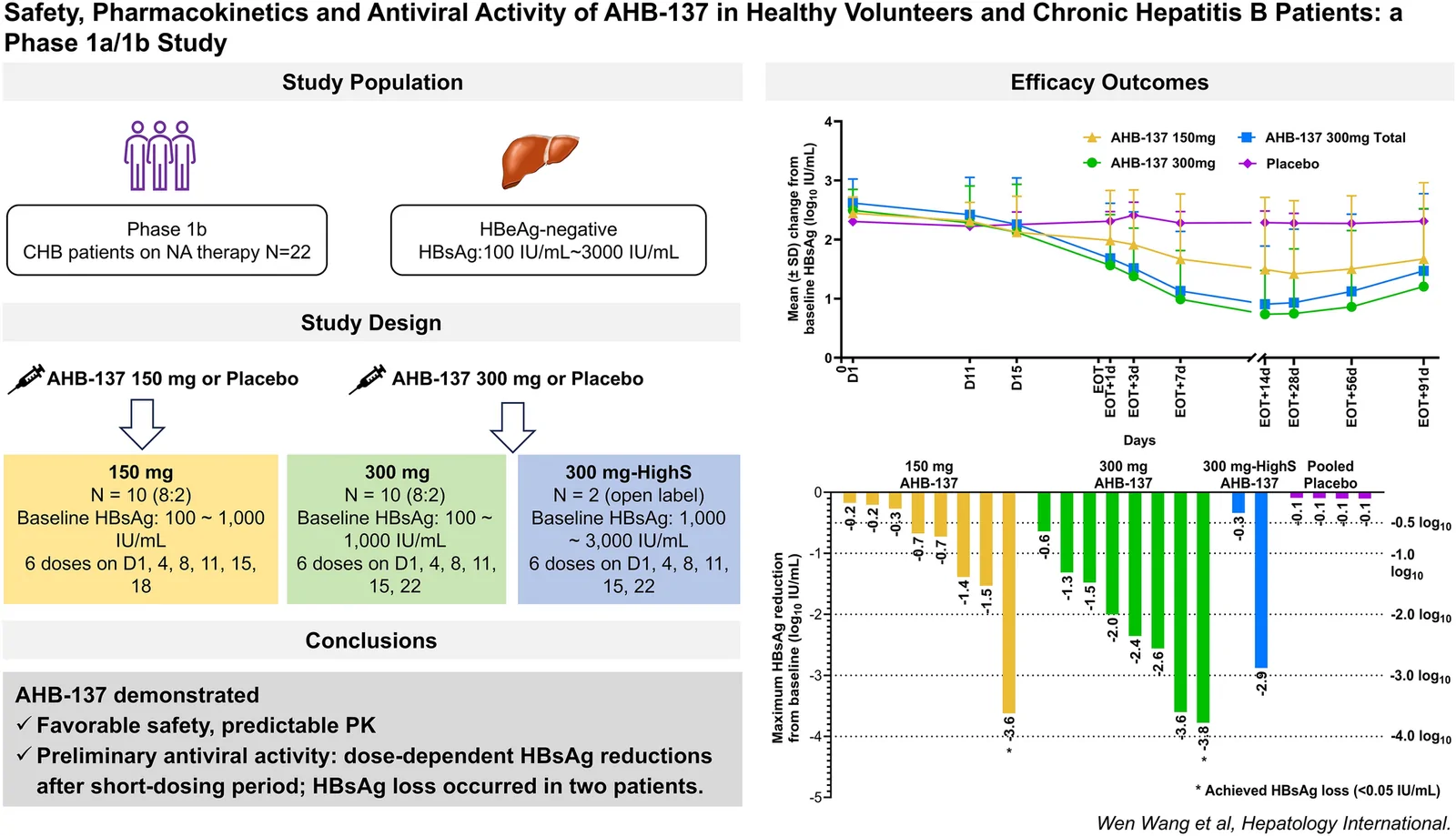

**Supplementary Information:**

The online version contains supplementary material available at 10.1007/s12072-026-11102-7.

## Introduction

Despite widespread vaccination programs, chronic hepatitis B (CHB) remains a major global health challenge, affecting approximately 258 million individuals worldwide and causing an estimated 1.1 million deaths annually [1–3]. China bears a substantial burden, with ~ 75 million people living with CHB [4], and increasing incidence of CHB-related cirrhosis and hepatocellular carcinoma (HCC) [5].

The goal of CHB treatment is functional cure, defined as sustained hepatitis B surface antigen (HBsAg) loss for at least 24 weeks off therapy with hepatitis B virus (HBV) DNA below the lower limit of quantification (LLOQ) [3, 6]. Current therapies include nucleos(t)ide analogues (NAs) and pegylated interferon (PEG-IFN). NAs effectively suppress HBV DNA but rarely achieve HBsAg loss (0–3% after long-term NA therapy), while IFN therapy is limited by safety and tolerability concerns [1, 3, 7–9].

Transcript-targeting antisense oligonucleotides (ASOs) have demonstrated clinical potential. Bepirovirsen has shown sustained HBsAg loss in approximately 9–10% of participants after 24 weeks of finite treatment [10, 11].

AHB-137 is a novel, unconjugated 2′-methoxyethyl (MOE)-modified ASO targeting a 20-nucleotide sequence in the highly conserved 3'-terminal region of HBV mRNA across genotypes. Preclinical studies demonstrated a favorable safety profile and potent antiviral activity against HBV transcripts derived from both cccDNA and integrated genomes, especially in reducing serum HBsAg [12, 13].

Based on these findings, a phase 1a/1b study was conducted in mainland China in parallel with a global phase 1 study (NCT05717686). Here, we report safety, tolerability, and PK of AHB-137 in Chinese healthy volunteers, as well as preliminary antiviral activity in Chinese NA-treated HBeAg-negative CHB patients.

## Materials and methods

### Study design and oversight

AB-10-8002 (ClinicalTrials.gov: NCT06115993) is a multi-part clinical study of AHB-137 conducted in China, comprising: (i) a phase 1a part in Chinese healthy volunteers, (ii) a phase 1b part in HBeAg-negative CHB patients on stable NA therapy, and (iii) a proof-of-concept phase 1c/2a part in NA-treated HBeAg-negative CHB patients. Here we reported only the prespecified analyses from phase 1a/1b and phase 1c/2a is ongoing and will be analyzed and reported separately. The overall phase 1 study design is provided in Supplementary Fig. 1.

Phase 1a was a single-site, randomized, double-blinded, placebo-controlled study evaluating the safety, tolerability, and PK of AHB-137 following single ascending doses (SAD) and multiple ascending doses (MAD) in healthy volunteers. Phase 1b consisted of a randomized, double-blinded, placebo-controlled dose escalation stage, followed by an open-label dose expansion stage to evaluate the safety, tolerability, PK and antiviral activity in NA-treated HBeAg-negative CHB patients. The first participant was enrolled on 04 August 2023, and the last phase 1b participant visit occurred on 21 June 2024.

The study was conducted in accordance with the Declaration of Helsinki and ICH Good Clinical Practice guidelines, with approval from relevant ethics committees in China. All participants provided written informed consent.

### Study participants

Eligible participants for phase 1a were healthy adults aged 18–55 years with body mass index (BMI) 18–28 kg/m^2^ and negative screening tests for HBV (HBsAg), hepatitis C virus, human immunodeficiency virus and treponema pallidum.

Eligible participants for phase 1b were adults aged 18–65 years with documented CHB, defined as HBsAg positivity for ≥ 6 months, HBeAg negative status, ALT ≤ 2 × upper limit of normal (ULN), total bilirubin < 2 × ULN, HBV DNA < 100 IU/mL, and stable NA monotherapy (tenofovir disoproxil fumarate, tenofovir alafenamide, or entecavir) for ≥ 6 months before screening. Key exclusion criteria included hepatitis C or D coinfection, human immunodeficiency virus infection, clinically significant liver fibrosis (e.g., liver stiffness of ≥ 12.4 kPa), and hepatocellular carcinoma. Full inclusion and exclusion criteria are listed in the Supplementary Methods.

### Study procedures and interventions

In phase 1a SAD, participants were randomized within each cohort (planned N = 8) to receive a single subcutaneous dose of AHB-137 (75, 150, 300, or 450 mg) or placebo (6:2) with sentinel dosing. In Phase 1a MAD, participants in 2 cohorts (planned N = 10) received 6 subcutaneous doses of AHB-137 (150 or 300 mg) over 4 weeks (Days 1, 4, 8, 11, 15, and 22, including loading doses on Days 4 and 11) or placebo (8:2) with sentinel dosing. Dose selection (150 and 300 mg) for the MAD cohorts was based on preclinical efficacy, PK, toxicology, and observed tolerability of 75–300 mg single doses. Preclinical efficacy translated to a human equivalent dose (240–280 mg) supported selection of 300 mg, while 150 mg was included to assess dose–response, and carried forward into phase 1b MAD to evaluate activity across the predicted efficacious range.

In Phase 1b dose escalation MAD, NA-treated HBeAg-negative CHB patients with baseline HBsAg > 100 to ≤ 1000 IU/mL (planned N = 10 per cohort) received 6 subcutaneous doses of AHB-137 (150 or 300 mg) over 4 weeks (Days 1, 4, 8, 11, 15, and 18) with loading doses on Days 4 and 11. In phase 1b expansion cohort, patients with baseline HBsAg > 1000 to ≤ 3000 IU/mL received open-label AHB-137 300 mg on Days 1, 4, 8, 11, 15, and 22, including loading doses on Days 4 and 11, with follow-up through Day 113. Detailed dosing procedures and escalation rules, the rationale for the loading dose schedule (Days 4 and 11), and dose selections are provided in the Supplementary Methods.

### Endpoints and assessments

Primary objectives of phase 1a were safety, tolerability and PK; for phase 1b, safety and tolerability were primary with second objective including preliminary antiviral activity and PK.

Safety assessments included treatment-emergent adverse events (TEAEs), serious adverse events (SAEs) and clinically significant abnormalities from laboratory measurements, vital signs and electrocardiograms (ECGs). TEAEs were defined as adverse events (AEs) occurring or worsening in severity after the first dose of study drug. Adverse events were graded using the Common Terminology Criteria for Adverse Events Version 5.0 (CTCAE V5.0) [14]; injection-site reactions (ISRs) were evaluated based on ISR reaction evaluation form adapted from FDA Preventative Vaccine Clinical Trial Toxicity Grading Scale [15].

Efficacy assessments included changes from baseline in HBsAg and HBV DNA, and incidence of hepatitis B surface antibody and HBeAg. HBsAg was quantified using the Elecsys HBsAg II Quant II assay (Roche Diagnostics) on the cobas e601 platform, with the LLOQ of 0.05 IU/mL.

Plasma PK endpoints included time to maximum plasma concentration (T_max_), maximum plasma concentration (C_max_), area under the plasma concentration–time curve (AUC_0–t_, AUC_0–∞_), terminal elimination half-life (t_1/2_), apparent clearance (CL/F), and apparent terminal volume of distribution (V_z_/F). For multiple dosing cohorts, minimum plasma concentration (C_min_) and accumulation ratios based on C_max_ (ARC_max_) and AUC (AR_AUC_) were also assessed. Urinary PK assessments included the amount of drug excreted in urine (Ae), the cumulative fraction of dose of drug excreted in urine (Fe), and renal clearance (CLr) in selected cohorts. Sampling schedules are provided in the Supplementary Methods.

### Statistical analysis

No formal sample-size calculation was performed. All participants receiving ≥ 1 dose with ≥ 1 postbaseline assessment were included in analyses sets. Statistical analyses were descriptive and summaries were presented by cohort and treatment groups. Continuous variables were summarized using descriptive statistics (n, mean, standard deviation [SD], median, minimum, and maximum), and categorical variables were summarized using counts and percentages.

CHB patients from the phase 1b dose expansion cohort who rolled over into the phase 1c/2a part were excluded from phase 1b analyses.

## Results

### Patient demographics and disposition

A total of 162 Chinese healthy volunteers were screened for phase 1a, of whom 52 were enrolled (SAD, n = 32; MAD, n = 20). In phase 1b, 70 NA-treated HBeAg-negative CHB patients were screened and 22 were enrolled and dosed, including 20 patients with baseline HBsAg ≤ 1000 IU/mL in dose escalation cohorts and 2 patients with baseline HBsAg > 1000 to ≤ 3000 IU/mL in the dose expansion cohort (300 mg-HighS; Supplementary Fig. 2). All these dosed participants (n = 74) completed the planned dosing and follow-up, except one placebo recipient in the phase 1a MAD 150 mg cohort who was lost to follow-up at the final visit (Day 113).

Baseline characteristics of healthy volunteers were comparable across cohorts (Supplementary Table 1). CHB patients’ characteristics were also balanced across cohorts (Table 1): patients were predominantly male (68.2%), with the mean (SD) age of 47.8 (7.2) years and BMI of 25.6 (3.1) kg/m^2^. Most had HBV genotype C (81.8%), baseline HBV DNA < LLOQ and ALT < ULN (95.5%), with mean (SD) HBsAg of 2.5 (0.3) log_10_ IU/mL. Patients had long-standing HBV infection (mean duration as 15.6 years) and were on stable NA therapy, primarily entecavir (77.3%) or tenofovir (13.6%).

**Table 1:** Demographic and baseline characteristics in NA-treated HBeAg-negative CHB patients

	Phase 1b—NA-treated HBeAg-negative CHB patients					
	150 mg	300 mg	300 mg-HighS	Pooled 300 mg	Total AHB-137	Pooled placebo
	N = 8	N = 8	N = 2	N = 10	N = 18	N = 4
Age (years), mean (SD)	52.4 (6.7)	43.3 (6.0)	50.5 (0.7)	44.7 (6.1)	48.1 (7.3)	46.5 (7.7)
Male, n (%)	6 (75.0)	5 (62.5)	0	5 (50.0)	11 (61.1)	4 (100)
Weight (kg), mean (SD)	71.7 (17.0)	75.3 (15.1)	65.8 (0.6)	73.4 (13.9)	72.6 (14.9)	68.1 (4.9)
BMI (kg/m^2^), mean (SD)	25.0 (3.4)	26.9 (3.8)	24.0 (1.4)	26.3 (3.6)	25.7 (3.5)	25.3 (0.5)
Baseline ALT (U/L), mean (SD)	30.3 (25.9)	26.0 (14.1)	23.9 (1.9)	25.6 (13.1)	27.7 (19.3)	26.5 (15.3)
HBV genotype						
B	1 (12.5)	2 (25.0)	0	2 (20.0)	3 (16.7)	1 (25.0)
C	7 (87.5)	6 (75.0)	2 (100)	8 (80.0)	15 (83.3)	3 (75.0)
Fibro Scan LSM/E	5.8 (1.8)	5.5 (1.4)	6.6 (0.9)	5.7 (1.3)	5.7 (1.5)	5.8 (1.7)
HBsAg (log_10_) (IU/ml)	2.4 (0.3)	2.5 (0.4)	3.1 (0.1)	2.6 (0.4)	2.5 (0.4)	2.3 (0.2)
HBV DNA, n (%)						
≥ LLOQ	0	0	1 (50.0)	1 (10.0)	1 (5.6)	0
Current antiviral treatment (NA drugs), n (%)						
ETV	8 (100)	4 (50.0)	2 (100)	6 (60.0)	14 (77.8)	3 (75.0)
TAF	0	3 (37.5)	0	3 (30.0)	3 (16.7)	0
TDF	0	1 (12.5)	0	1 (10.0)	1 (5.6)	1 (25.0)
Duration of hepatitis B (years), n (%)	17.7 (9.8)	11.6 (12.5)	22.2 (2.8)	13.7 (12.0)	15.4 (1.0)	16.4 (15.4)
NA therapy duration (years), mean (SD)	7.7 (5.3)	4.0 (2.8)	1.0 (0.2)	3.4 (2.8)	5.3 (4.5)	5.6 (6.0)

*ALT* alanine aminotransferase, *BMI* body mass index, *CHB* chronic hepatitis B, *ETV* entecavir, *HBsAg* hepatitis B surface antigen, *HBeAg* hepatitis B e antigen, *HBV* hepatitis B virus, *LLOQ* lower limit of quantification, *LSM* liver stiffness measurement, *NA* nucleos(t)ide analogue, *SD* standard deviation, *TAF* tenofovir alafenamide, *TDF* tenofovir disoproxil fumarate

### Safety and tolerability

AHB-137 was well tolerated in both healthy volunteers and NA-treated HBeAg-negative CHB patients. No death, SAEs, or TEAE leading to drug interruption or discontinuation occurred (Table 2, Supplementary Table 2). TEAEs were reported in 69.2% (36/52) of healthy volunteers and 86.4% (19/22) of CHB patients, and treatment-related emergent adverse events (TRAEs) were reported in 65.3% (34/52) and 72.7% (16/22), respectively. Most TRAEs were Grade 1–2, reversible and resolved by end of study. The most common TRAEs included ISRs (28.1–75.0%), pyrexia (10.0–27.3%), and asymptomatic lab abnormalities (e.g., C-reactive protein [CRP] increased [36.4–50.0%], lymphocyte count decreased [27.3–31.3%]) (Supplementary Table 5–6). Grade ≥ 3 TRAEs occurred in 13.5% (7/52) of healthy volunteers and 18.2% (4/22) of CHB patients and were limited to self-limiting laboratory abnormalities (including lymphocytes count decreased, blood creatine phosphokinase increased, ALT increased, AST increased, and GGT increased). ISRs (injection site reaction, pain, erythema, swelling, and induration) were all Grade 1–2, transient, resolved during the study, and showed no apparent dose relationship (Supplementary Table 7–8).

**Table 2:** Overview of treatment-emergent adverse events in NA-treated HBeAg-negative CHB patients

	Phase 1b—NA-treated HBeAg-negative CHB patients					
	150 mg	300 mg	300 mg-HighS	Pooled 300 mg	Total AHB-137	Pooled placebo
	N = 8	N = 8	N = 2	N = 10	N = 18	N = 4
Any TEAE	8 (100)	8 (100)	2 (100)	10 (100)	18 (100)	1 (25.0)
Any TRAE	7 (87.5)	7 (87.5)	2 (100)	9 (90.0)	16 (88.9)	0
Any SAE	0	0	0	0	0	0
Any TEAE leading to drug interruption/discontinuation/ death	0	0	0	0	0	0
TRAE by worst severity						
Grade 1	5 (62.5)	2 (25.0)	0	2 (20.0)	7 (38.9)	0
Grade 2	2 (25.0)	2 (25.0)	1 (50.0)	3 (30.0)	5 (27.8)	0
≥ Grade 3	0	3 (37.5)	1 (50.0)	4 (40.0)	4 (22.2)	0
Grade 3	0	3 (37.5)	1 (50.0)	4 (40.0)	4 (22.2)	0
Grade 4	0	0	0	0	0	0
Injection site reaction	7 (87.5)	6 (75.0)	1 (50.0)	7 (70.0)	14 (77.8)	0

*CHB* chronic hepatitis B, *SAE* serious adverse event, *TEAE* treatment-emergent adverse event, *TRAE* treatment-related adverse event

**Fig. 1:**
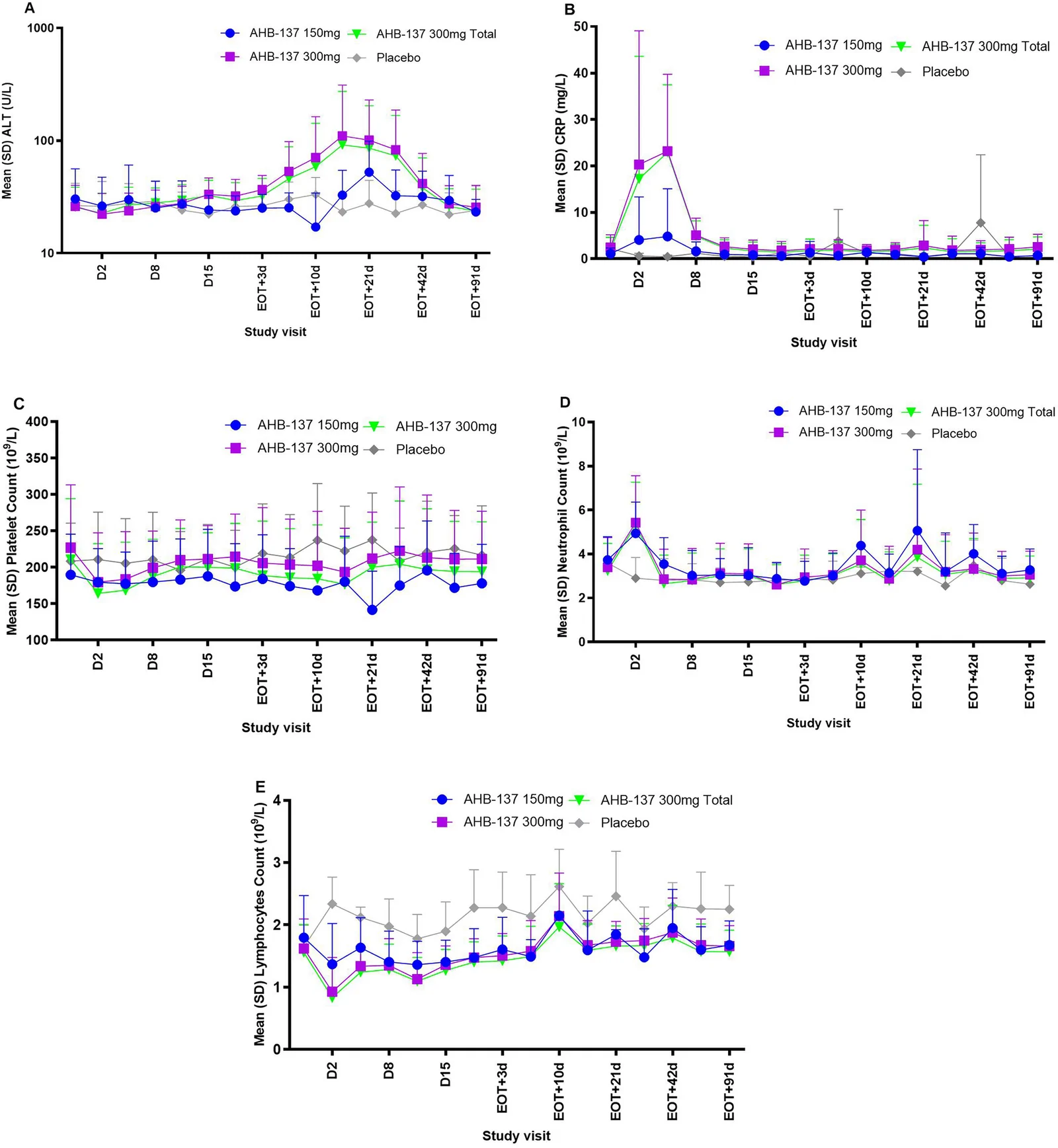
Evaluations of selected laboratory parameters (ALT [**A**], CRP [**B**], platelet [**C**], neutrophil count [**D**], and lymphocytes count [**E**]) overtime during phase 1b–CHB patients receiving multiple doses of AHB-137 (150, 300 mg) or placebo. AHB-137 300 mg total cohort consisted of dose escalation 300 mg cohort and dose expansion 300 mg cohort. *ALT* alanine aminotransferase, *CHB* chronic hepatitis B, *CRP* C-reactive protein

No obvious clinically meaningful changes were observed in vital signs, ECGs, urinalysis, or most hematology parameters. Transient CRP increased, lymphocytes decreased, and neutrophil increased were observed after the first dose of AHB-137 in some participants, with peak value of CRP on around Days 2–4, nadir value of lymphocytes count on Day 2 and peak value of neutrophil count on Day 2, without associated symptoms (Fig. 1 and Supplementary Fig. 3). ALT increases were common (78.4% [58/74]) but predominantly Grade 1–2 (Fig. 1 and Supplementary Fig. 3).

One CHB patient in the phase 1b dose escalation 300 mg cohort developed a Grade 3 ALT elevation, peaking after end of treatment (EOT; 3.2 × ULN at Day 29 and 12.1 × ULN at Day 36) and coinciding with a rapid HBsAg reduction and HBsAg loss (< 0.05 IU/mL). The event resolved spontaneously with no impact on liver function (bilirubin, coagulation).

### Antiviral activity

**Fig. 2:**
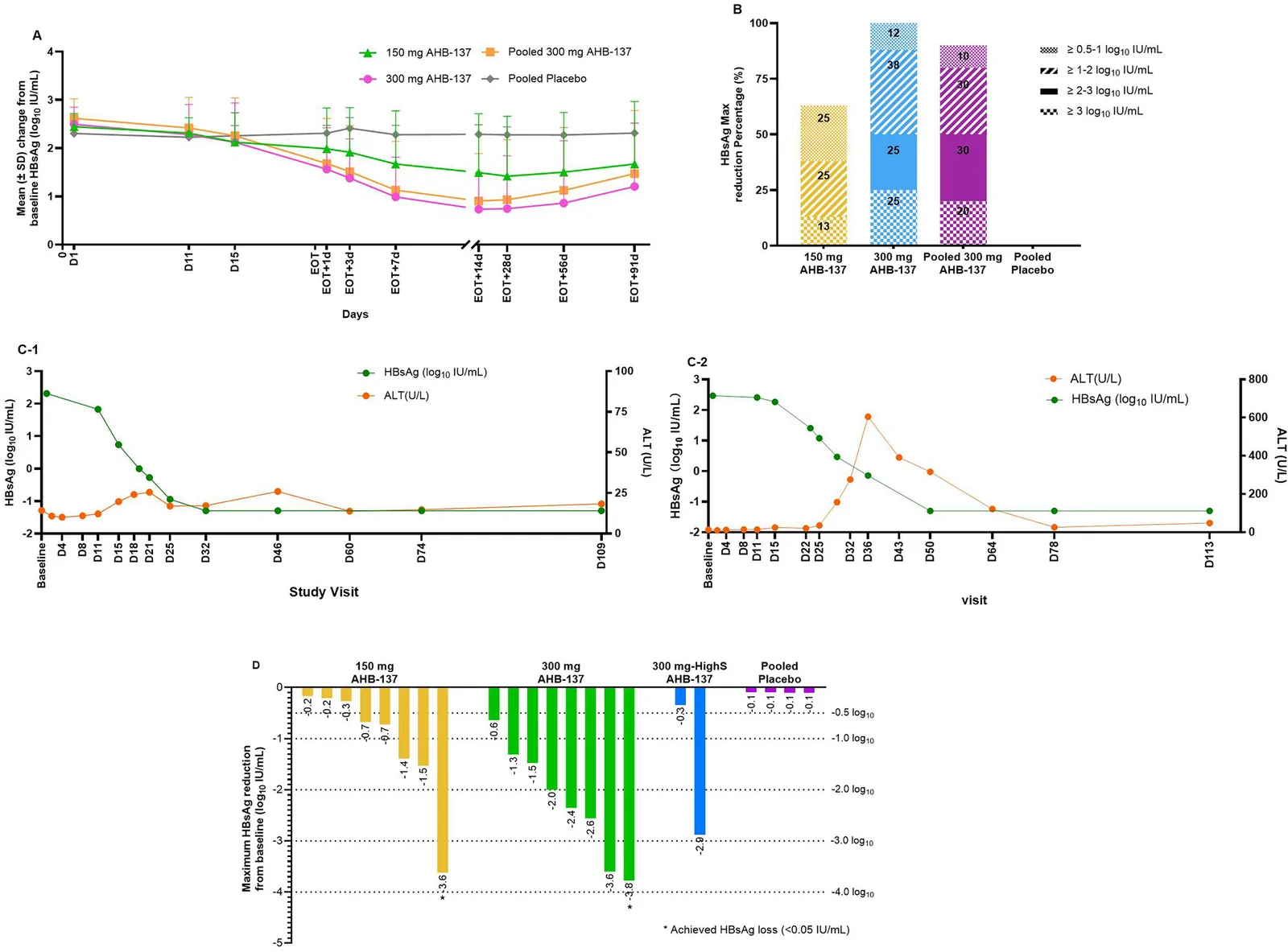
Antiviral Activity of AHB-137. **A** Mean (± SD) HBsAg (log_10_ IU/mL) change from baseline over time; **B** categorical changes from baseline in HBsAg; **C1-2** individual examples of HBsAg and ALT change, including a representative participant illustrating the temporal relationship between ALT elevation and HBsAg decline (C2); **D** maximum HBsAg reduction from baseline in individual CHB patients through end of study (last visit). *CHB* chronic hepatitis B, *HBsAg* hepatitis B surface antigen, *SD* standard deviation

Following 4 weeks of dosing (6 doses), serum HBsAg levels exhibited a rapid dose-dependent decline in CHB patients with AHB-137 150 mg and 300 mg, with the median nadir change from baseline observed after EOT (Days 29–50) (Fig. 2). The mean (SD) maximum HBsAg reduction was -1.1 (1.2), -2.1 (1.2), and -0.1 (0.0) log_10_ IU/mL in the 150 mg, pooled 300 mg, and pooled placebo groups, respectively. Among patients with baseline HBsAg < 1000 IU/mL, 87.5% (7/8) and 50.0% (4/8) in the 300 mg group achieved maximum HBsAg reductions > 1 and > 2 log_10_ IU/mL, respectively, compared with 37.5% (3/8) and 12.5% (1/8) in the 150 mg group (Table 3).

**Table 3:** Summary of preliminary efficacy for NA-treated HBeAg-negative CHB patients (Phase 1b)

	Phase 1b—NA-treated HBeAg-negative CHB patients					
	150 mg	300 mg	300 mg-HighS	Pooled 300 mg	Total AHB-137	Pooled placebo
	N = 8	N = 8	N = 2	N = 10	N = 18	N = 4
Baseline HBsAg (log_10_ IU/mL), mean (SD)	2.4 (0.3)	2.5 (0.4)	3.1 (0.1)	2.6 (0.4)	2.5 (0.4)	2.3 (0.2)
HBsAg maximum reduction log_10_ IU/mL (mean [SD])	− 1.1 (1.2)	− 2.22 (1.1)	− 1.6 (1.8)	− 2.1 (1.2)	− 1.6 (1.2)	− 0.1 (0.0)
HBsAg maximum reduction (%)						
≥ 0.5 log_10_ IU/mL	5 (62.5)	8 (100.0)	1 (50.0)	9 (90.0)	14 (77.8)	0
≥ 1 log_10_ IU/mL	3 (37.5)	7 (87.5)	1 (50.0)	8 (80.0)	11 (61.1)	0
≥ 2 log_10_ IU/mL	1 (12.5)	4 (50.0)	1 (50.0)	5 (50.0)	6 (33.3)	0
≥ 3 log_10_ IU/mL	1 (12.5)	2 (25.0)	0	2 (20.0)	3 (16.7)	0
HBsAg loss (< 0.05 IU/mL)	1 (12.5)	1 (12.5)	0	1 (10.0)	2 (11.1)	0
HBV DNA (< LLOQ)	8 (100.0)	8 (100.0)	2 (100.0)	10 (100.0)	18 (100.0)	4 (100.0)
HBV DNA (< LLOQ) and HBsAg (< 0.05 IU/mL), n (%)	1 (12.5)	1 (12.5)	0	1 (10.0)	2 (11.1)	0

*CHB* chronic hepatitis B, *HBsAg* hepatitis B surface antigen, *HBV DNA* hepatitis B virus DNA, *LLOQ* lower limit of quantitation, *SD* standard deviation

HBsAg loss was observed in 2 patients (one each in the dose escalation 150 mg and 300 mg cohorts), first detected at post-treatment visits (Day 32 [14 days after EOT] and Day 50 [28 days after EOT], respectively) and maintained through follow up (91 days after EOT). No HBsAg loss was observed in the 300 mg dose-expansion cohort (Fig. 2D). Given the limited sample size and variability across cohorts, these findings should be interpreted with caution.

No HBsAg seroconversion was observed. HBV DNA remained suppressed (< LLOQ) in all AHB-137 treated CHB patients (n = 18), including one patient with quantifiable HBV DNA at baseline (≥ LLOQ to < 100 IU/mL) who achieved HBV DNA < LLOQ post treatment (Table 3).

### Pharmacokinetics

#### Plasma PK analysis

Plasma PK was evaluated in 52 healthy volunteers and 22 NA-treated HBeAg-negative CHB patients; mean concentration–time profiles are shown in Supplementary Fig. 4. Overall, PK characteristics were comparable between healthy volunteers and CHB patients. Following single (75–450 mg) and multiple (150 and 300 mg) subcutaneous dosing, AHB-137 was rapidly absorbed, with median T_max_ of approximately 3.5–5.0 h in healthy volunteers and 3.0–3.5 h in CHB patients (Table 4). Systemic exposure (AUC and C_max_) increased approximately dose-proportionally after single dosing (β_1_ slope estimates close to 1, Supplementary Table 9).

**Table 4:** Plasma PK parameters for healthy volunteers and CHB patients (pharmacokinetic parameter set)

Geomean (%CV)^a^	1a-healthy volunteers-SAD				1a-healthy volunteers-MAD				1b-CHB patients-MAD					
									Dose escalation				Dose expansion	
	75 mg N = 6	150 mg N = 6	300 mg N = 6	450 mg N = 6	150 mg D1 N = 8	150 mg D22 N = 8	300 mg D1 N = 8	300 mg D22 N = 8	150 mg D1 N = 8	150 mg D18 N = 8	300 mg D1 N = 8	300 mg D22 N = 8	300 mg-HighS D1 N = 2	300 mg-HighS D22 N = 2
T_max_ (h)	5.0 (2.0, 8.0)	4.0 (3.0, 4.0)	4.0 (3.0, 6.0)	3.5 (1.5, 6.0)	4.0 (4.0, 6.0)	4.0 (2.0, 4.0)	4.0 (3.0, 4.0)	3.5 (2.0, 6.0)	3.5 (3.0, 4.0)	3.0 (3.0, 4.0)	3.5 (3.0, 4.0)	3.0 (3.0, 4.0)	3.5 (3.0, 4.0)	3.0 (3.0, 3.0)
C_max_ (nmol/L)	215 (25.7)	555 (43.1)	1160 (29.0)	1530 (24.5)	596 (54.9)	524 (42.8)	1020 (14.8)	840 (15.8)	492 (75.4)	366 (71.5)	874 (35.7)	878 (25.7)	867 (17.3)	815 (1.6)
AUC_0–last_ (nmol*h/L)	2210 (22.9)	5470 (35.7)	12,400 (27.3)	17,400 (18.2)	5720 (39.1)	5770 (34.6)	11,100 (10.9)	10,700 (12.0)	4950 (47.5)	4750 (45.8)	10,100 (19.6)	10,600 (19.8)	10,500 (1.1)	9760 (14.5)
t_1/2_ (h)^b^	2.9 (2.2, 8.0)	12.9 (6.5, 99.8)	104 (77.9, 136)	84.2 (54.5, 131)	6.6 (4.1, 19.8)	128 (77.4, 273)	6.5 (6.2, 7.1)	195 (120, 267)	6.5 (4.5, 14.2)	145 (102, 479)	6.9 (6.4, 17.7)	129 (20.7, 245)	7.1 (6.8, 7.4)	168 (149, 187)
CL/F (L/h)^b^	4.7 (22.6)	3.8 (36.0)	3.2 (28.4)	3.6 (17.9)	3.8 (41.1)	3.8 (30.1)	3.8 (10.9)	3.8 (11.2)	4.2 (47.5)	3.8 (37.8)	4.1 (19.7)	3.9 (19.4)	3.9 (1.0)	4.2 (13.7)
AR_AUC_	NA	NA	NA	NA	NA	0.95 (9.9)	NA	0.89 (11.0)	NA	0.89 (8.3)	NA	0.99 (9.6)	NA	0.88 (16.4)
ARC_max_	NA	NA	NA	NA	NA	0.88 (31.1)	NA	0.83 (20.3)	NA	0.74 (30.9)	NA	1.01 (32.1)	NA	0.94 (19.0)

Enrolled CHB patients in phase 1b were allocated into dose escalation and dose expansion cohorts with different baseline HBsAg level (dose escalation 150 mg and 300 mg cohorts: 100 IU/mL ≤ HBsAg ≤ 1000 IU/mL; dose expansion 300 mg-HighS cohort: 1000 IU/mL < HBsAg ≤ 3000 IU/mL)

*AR* accumulation ratio, *AR*_*AUC*_ accumulation ratio for AUC (AUC_0–72h_ on D22/AUC_0–72h_ on D1), *AR*_*Cmax*_ accumulation ratios for *C*_max_ (*C*_max_ on D22/*C*_max_ on D1), *AUC*_*0-last*_ area under the plasma concentration–time curve from time zero to the last measurable concentration, *AUC*_*0-inf*_ area under the plasma concentration–time curve from time zero to infinity, *C*_*max*_ maximum concentration, *CL/F* apparent clearance, *CV* coefficient of variation, *D(X)* Day X, *n* number of participants with calculable PK parameters, *N* participant count, *NA* not applicable, *T*_*max*_ time to maximum concentration, *t*_*1/2*_ half-life

^a^Data are median (min, max) for T_max_ and t_1/2_

^b^n = 5 for 1a-SAD 300 mg dose group; n = 7 for 1a-MAD 150 mg D1 and D22; n = 7 for 1b-MAD 150 mg D22. T_last_ was 24–337h in Ia-SAD; T_last_ was 48–72 h (D1) and 168–696 h (D22) in 1a-MAD and 1b-MAD

After multiple dosing, no clinically meaningful accumulation was observed. In healthy volunteers, AR_AUC_ values were 0.95 and 0.89, and ARC_max_ values were 0.88 and 0.83 for the 150 mg and 300 mg cohorts, respectively. Similar findings were observed in CHB patients (AR_AUC_ of 0.878–0.890, ARC_max_ of 0.743–1.01), in consistent PK across populations.

#### Urine PK analysis

Renal excretion of full-length AHB-137 was minimal across dose levels. After single or first dosing, urinary excretion increased slightly with dose but remained low (geometric mean Fe_0–72 h_ < 2%, Fe_0–168 h_ < 4% on Day 1, Supplementary Table 10). After multiple dosing, higher cumulative urinary excretion was observed (Fe_0–72 h_ of 5–8%, Fe_0–168 h_ of 11–15% on Day 22, Supplementary Table 10), with corresponding increases in renal clearance, reflecting accumulation of prior doses. Overall, urinary excretion was a minor route of elimination for full-length AHB-137.

## Discussion

Given the persistent unmet need for finite, functional cure-oriented therapies in CHB, ASOs represent a promising strategy to achieve deeper HBsAg reduction and potentially facilitate immune restoration [10, 16]. This phase 1a/1b study provides the first clinical characterization of AHB-137 in participants of mainland China and demonstrated a favorable safety profile, and predictable PK, and rapid, dose-dependent HBsAg reductions in NA-treated HBeAg-negative CHB patients. Preliminary HBsAg loss observed in a small subset of patients. The phase 1b population was broadly representative of real-world CHB patients in China and aligned with populations targeted in guideline-recommended functional cure strategies [10, 17–19].

AHB-137 was generally well tolerated, with no deaths, SAEs, or discontinuations. Most TRAEs were Grade 1–2, reversible and resolved by end of study. Common TRAEs included ISRs, pyrexia, transient, and asymptomatic laboratory abnormalities, e.g., CRP increases, lymphocyte decreases, neutrophil increases and low-grade transaminase elevations, which were broadly consistent with class effects of second-generation ASOs, including bepirovirsen [10, 20, 21]. Grade ≥ 3 TRAEs were limited to self-limiting laboratory abnormalities and did not require treatment interruption or discontinuation. ALT elevations were common but predominantly Grade 1–2 and reversible.

One CHB patient in the 300 mg cohort developed a Grade 3 ALT elevation (peak 604.9 U/L; 12.1 × ULN) following dosing, coinciding with rapid HBsAg decline and subsequent HBsAg loss (from 293 IU/mL at baseline to < 0.05 IU/mL by Day 50; Fig. 2C-2). This event was not accompanied by bilirubin elevation, coagulation abnormalities (e.g., INR), or evidence of impaired hepatic synthetic function. The event was assessed by the investigator as related to study treatment and resolved. The temporal association with antigen decline and absence of hepatic dysfunction are consistent with an immune-mediated flare rather than drug-induced liver injury, as reported with other HBV-targeting therapies, including bepirovirsen [10, 11]. Continued monitoring of liver function will be important in future studies, particularly with longer treatment duration and combination strategies. Transient CRP increases and lymphocyte decreases were observed following AHB-137 dosing and are considered consistent with class-related effects of ASOs, likely reflecting mild innate immune activation rather than being primarily related to the subcutaneous route of administration, as reported for other HBV-targeting ASOs, including bepirovirsen [11]. No oligonucleotide-class associated effects, including clinically significant thrombocytopenia, complement activation, or renal impairment, were observed throughout the study [11, 22].

AHB-137 induced rapid, dose-dependent HBsAg reductions after a short 4-week dosing period. Two CHB patients achieved HBsAg loss during follow up, both with baseline HBsAg ≤ 1000 IU/mL, whereas no HBsAg loss was observed in the higher baseline cohort. Given the limited sample size and variability across cohorts, these findings should be interpreted with caution. These observations align with prior data for bepirovirsen [10, 23], which also suggest that lower baseline HBsAg (≤ 1000 IU/mL) is associated with a higher likelihood of response.

In the present study, AHB-137 300 mg achieved a rapid reduction of 2.22 log_10_ IU/mL within 4 weeks, with variability in individual responses. In the phase 2 bepirovirsen study (NCT02981602), a mean reduction of ~ 1.99 log_10_ IU/mL at Day 29 was observed in NA-treated patients based on a small sample size (n = 5) and individual responses demonstrated variability [20]. These findings provide contextual reference for the magnitude and kinetics of HBsAg decline, while cross-trial comparisons should be interpreted with caution due to the differences in baseline HBsAg range, sample size, and study design.

AHB-137 and bepirovirsen share the same target sequence but differ in pharmacological structure. Bepirovirsen follows a conventional single-gap (5–10–5) gapmer architecture with a continuous central DNA gap flanked by terminal 2′-MOE modifications, whereas AHB-137 adopts a multi-segment structural design in which the gap is divided into shorter segments separated by modified regions, with additional site-specific 2′-modifications. These structural features may influence target binding affinity, resistance to nuclease-mediated degradation, and RNase H-mediated cleavage.

PK profiles were comparable between healthy volunteers and CHB patients, with rapid absorption (median T_max_ 3–5 h), approximately dose-proportional increases in exposure and no clinically meaningful accumulation (AR_AUC_ and ARC_max_ close to 1). Concentration–time profiles showed an early rapid decline(within the first 48 h post-dose) followed by a slower terminal phase at lower concentrations, consistent with prior experience of 2′-MOE ASOs and supportive of once-weekly administration [11, 24, 25]. Based on class experience, elimination is expected to be driven primarily by nuclease-mediated metabolism in tissues with urinary excretion mainly as chain-shortened metabolites rather than intact drug [11, 26]. Due to the limitation of LC–MS/MS assay in this study precluding detection of late-time-point plasma concentrations, the estimated median terminal elimination half-life (128–195 h) likely underestimates overall persistence and may not reflect the true hepatic retention; based on cynomolgus monkey preclinical data, the human hepatic half-life is predicted to be ~ 2 weeks (data not shown).

The study has limitations. Sample size was modest, and efficacy findings, including HBsAg loss, are subject to uncertainty and should be interpreted with caution. The dose-expansion cohort with baseline HBsAg > 1000 IU/mL was small due to rollover (n = 8) into an ongoing phase 1c/2a, limiting conclusions in higher-antigen subgroups. HBsAg loss was confined to CHB patients with lower baseline HBsAg, and no seroconversion occurred after the short 4-week treatment period, underscoring the need for longer treatment durations, especially in individuals with higher baseline HBsAg. These findings are preliminary and require confirmation in larger studies with long-term surveillance.

In conclusion, AHB-137 administrated subcutaneously (SAD up to 450 mg; MAD up to 300 mg) was safe and well tolerated in Chinese healthy volunteers and CHB patients, with predictable PK profile and preliminary antiviral activity. Ongoing phase 2 studies (NCT06550128, NCT06829329, NCT06993480, NCT07069569, NCT07146100) will further evaluate its clinical potential across broader populations, regimens, treatment durations, and combination strategies.

Previous Presentation Portions of these data were presented at the EASL Congress 2024 (Poster LBP‑043 and LBP‑019, Milan, Italy).

## Supplementary Information

Below is the link to the electronic supplementary material.

Supplementary file1 (DOCX 530 KB)

## Data Availability

De‑identified participant data and related documents will be made available upon reasonable request to the corresponding author after publication, subject to data‑sharing agreements.

## Abbreviations

AE: Adverse events
ALT: Alanine aminotransferase
AR: Accumulation ratio
ASO: Antisense oligonucleotide
AST: Aspartate aminotransferase
AUC: Area under the time-concentration curve
BMI: Body mass index
CHB: Chronic hepatitis B
CL/F: Apparent clearance
CRP: C-reactive protein
CTCAE: Common Terminology Criteria for Adverse Events
ECG: Electrocardiogram
MAD: Multiple ascending dose
HBsAg: Hepatitis B surface antigen
HBV: Hepatitis B virus
HCC: Hepatocellular carcinoma
IFN: Interferon
ISR: Injection-site reaction
LLOQ: Lower limit of quantification
NA: Nucleos(t)ide analogue
PK: Pharmacokinetic
SAD: Single ascending dose
siRNA: Small interfering RNA
TEAE: Treatment-emergent AE
TRAE: Treatment-related adverse event
ULN: Upper limit of the normal range
