# Supplementary material for: Safety, pharmacokinetics and antiviral activity of AHB‑137 in healthy volunteers and chronic hepatitis B patients: a phase 1a/1b study
Source: Hepatol Int. 2026 May 23;20(4):1035–46. doi: 10.1007/s12072-026-11102-7 (PMC13518476; doi:10.1007/s12072-026-11102-7)
Supplement: Supplementary file 1 [file 12072_2026_11102_MOESM1_ESM.docx]

**Original Article**

**Hepatology International**

**Title:** Safety, Pharmacokinetics and Antiviral Activity of AHB‑137 in Healthy Volunteers and Chronic Hepatitis B Patients: a Phase 1a/1b Study

**Authors:**

Wen Wang^1^, Jiajia Mai^1^, Jinlin Hou^2^, Zhongyuan Xu^2^, Xian Yu^3^, Hong Ren^3^, Xieer Liang^2^, Yi Yang^3^, Zhihong Liu^2^, Hong Zhang^1^, Shan Zhong^3^, Yilei Wen^4^, Tingting Lu^4^, Haixia Cao^4^, Xiao Qiu^4^, Lidan Wang^4^, Di Zhao^4^, Miao Wang^4^, Chengyong Yang^4,5^, Guofeng Cheng^4,5^, Junqi Niu^6*^ and Yanhua Ding^1*^

**Affiliations:**

^1^Phase I Clinical Trial Center, The First Hospital of Jilin University, No. 1 Xinmin Street, Changchun, Jilin, China.

^2^Nanfang Hospital, Southern Medical University, Guangzhou, China

^3^The Second Affiliated Hospital, Chongqing Medical University, Chongqing, China

^4^Ausper Biopharma Co. Ltd., Hangzhou, China.

^5^AusperBio Therapeutics Inc., Foster City, CA, USA.

^6^Department of Hepatology, Center of Infectious Diseases and Pathogen Biology, The First Hospital of Jilin University, Changchun, China.

*Corresponding Authors: Yanhua Ding, Phase I Clinical Trial Center, The First Hospital of Jilin University, No. 1 Xinmin Street, Changchun, Jilin Province, China. Email: dingyanh@jlu.edu.cn; Junqi Niu, Department of Hepatology, Center of Infectious Diseases and Pathogen Biology, The First Hospital of Jilin University, Changchun, China. Email: junqiniu@aliyun.com.

# Supplementary Information

## Supplementary Tables

**Supplementary Table 1: Demographic and baseline characteristics in healthy volunteers**

|  | **Phase 1a – Healthy Volunteers** | | | | | | | | | |
| --- | --- | --- | --- | --- | --- | --- | --- | --- | --- | --- |
|  | **SAD** | | | | | | **MAD** | | | |
|  | **75 mg** | **150 mg** | **300 mg** | **450 mg** | **Total AHB-137** | **Placebo** | **150 mg** | **300 mg** | **Total AHB-137** | **Placebo** |
|  | **N=6** | **N=6** | **N=6** | **N=6** | **N=24** | **N=8** | **N=8** | **N=8** | **N=16** | **N=4** |
| Age (years), mean (SD) | 44.7 (8.1) | 36.3 (9.9) | 37.5 (7.6) | 36.0 (6.7) | 38.6 (8.4) | 42.6 (8.7) | 39.1 (8.5) | 43.6 (7.1) | 41.4 (7.9) | 48.5 (7.2) |
| Male, n (%) | 4 (66.7) | 3 (50.0) | 3 (50.0) | 3 (50.0) | 13 (54.2) | 3 (37.5) | 4 (50.0) | 5 (62.5) | 9 (56.3) | 1 (25.0) |
| Ethnicity, n (%) |  |  |  |  |  |  |  |  |  |  |
| Han | 6 (100) | 6 (100) | 6 (100) | 6 (100) | 24 (100) | 8 (100) | 8 (100) | 8 (100) | 16 (100) | 4 (100) |
| Weight (kg), mean (SD) | 65.9 (8.8) | 64.8 (8.0) | 61.4 (11.2) | 62.8 (10.2) | 63.7 (9.2) | 64.8 (8.5) | 63.6 (15.8) | 70.4 (9.1) | 67.0 (13.0) | 58.0 (4.4) |
| BMI (kg/m^2^), mean (SD) | 24.2 (2.3) | 24.8 (2.6) | 23.7 (2.3) | 22.5 (2.6) | 23.8 (2.5) | 24.1 (1.5) | 23.3 (2.4) | 25.1 (1.9) | 24.2 (2.3) | 22.5 (1.7) |

Abbreviations: BMI, body mass index; MAD, multiple ascending dose; N, number of participants; NA, not applicable; SAD, single ascending dose; SD, standard deviation.

**Supplementary Table 2: Overview of treatment-emergent adverse events in healthy volunteers (Phase 1a)**

|  | **Phase 1a - Healthy Volunteers** | | | | | | | | | |
| --- | --- | --- | --- | --- | --- | --- | --- | --- | --- | --- |
| **AE Category, n (%)** | **SAD** | | | | | | **MAD** | | | |
|  | **75 mg** | **150 mg** | **300 mg** | **450 mg** | **Total AHB-137** | **Placebo** | **150 mg** | **300 mg** | **Total AHB-137** | **Placebo** |
|  | **N=6** | **N=6** | **N=6** | **N=6** | **N=24** | **N=8** | **N=8** | **N=8** | **N=16** | **N=4** |
| Any TEAE | 2 (33.3) | 3 (50.0) | 6 (100) | 6 (100) | 17 (70.8) | 2 (25.0) | 8 (100) | 8 (100) | 16 (100) | 1 (25.0) |
| Any TRAE | 1 (16.7) | 3 (50.0) | 6 (100) | 6 (100) | 16 (66.7) | 1 (12.5) | 8 (100) | 8 (100) | 16 (100) | 1 (25.0) |
| Any SAE | 0 | 0 | 0 | 0 | 0 | 0 | 0 | 0 | 0 | 0 |
| Any TEAE leading to drug interruption/ discontinuation/ death | 0 | 0 | 0 | 0 | 0 | 0 | 0 | 0 | 0 | 0 |
| TRAE by Worst Severity | | | | | | | | | | |
| Grade 1 | 1 (16.7) | 2 (33.3) | 1 (16.7) | 0 | 4 (16.7) | 1 (12.5) | 3 (37.5) | 1 (12.5) | 4 (25.0) | 1 (25.0) |
| Grade 2 | 0 | 0 | 5 (83.3) | 1 (16.7) | 6 (25.0) | 0 | 4 (50.0) | 7 (87.5) | 11 (68.8) | 0 |
| ≥ Grade 3 | 0 | 1 (16.7) | 0 | 5 (83.3) | 6 (25.0) | 0 | 1 (12.5) | 0 | 1 (6.3) | 0 |
| Grade 3 | 0 | 1 (16.7) | 0 | 2 (33.3) | 3 (12.5) | 0 | 1 (12.5) | 0 | 1 (6.3) | 0 |
| Grade 4 | 0 | 0 | 0 | 3 (50.0) | 3 (12.5) | 0 | 0 | 0 | 0 | 0 |
| Injection site reaction | 1 (16.7) | 2 (33.3) | 4 (66.7) | 2 (33.3) | 9 (37.5) | 0 | 7 (87.5) | 8 (100) | 15 (93.8) | 0 |

Abbreviations: MAD, multiple ascending dose; SAD, single ascending dose; SAE, serious adverse event; TEAE, treatment-emergent adverse event; TRAE, treatment-related adverse event.

**Supplementary Table 3: Treatment-emergent adverse events reported in ≥2 healthy** **volunteers in any treatment cohort by SOC and PT (Phase 1a)**

|  | **Phase 1a - Healthy Volunteers** | | | | | | | | | |
| --- | --- | --- | --- | --- | --- | --- | --- | --- | --- | --- |
| **System Organ Class/**  **Preferred Term, n (%)** | **SAD** | | | | | | **MAD** | | | |
|  | **75 mg** | **150 mg** | **300 mg** | **450 mg** | **Total AHB-137** | **Placebo** | **150 mg** | **300 mg** | **Total AHB-137** | **Placebo** |
|  | **N=6** | **N=6** | **N=6** | **N=6** | **N=24** | **N=8** | **N=8** | **N=8** | **N=16** | **N=4** |
| Participants with at least one TEAE | 0 | 2 (33.3) | 6 (100) | 6 (100) | 14 (58.3) | 1 (12.5) | 8 (100) | 8 (100) | 16 (100) | 1 (25.0) |
| **Investigations** | **0** | **1 (16.7)** | **5 (83.3)** | **6 (100)** | **12 (50.0)** | **1 (12.5)** | **5 (62.5)** | **7 (87.5)** | **12 (75.0)** | **1 (25.0)** |
| C-reactive protein increased | 0 | 1 (16.7) | 5 (83.3) | 6 (100) | 12 (50.0) | 0 | 5 (62.5) | 5 (62.5) | 10 (62.5) | 1 (25.0) |
| Lymphocyte count decreased | 0 | 0 | 4 (66.7) | 6 (100) | 10 (41.7) | 0 | 2 (25.0) | 4 (50.0) | 6 (37.5) | 0 |
| Neutrophil count increased | 0 | 0 | 2 (33.3) | 4 (66.7) | 6 (25.0) | 0 | 0 | 1 (12.5) | 1 (6.3) | 0 |
| Alanine aminotransferase increased | 0 | 0 | 2 (33.3) | 1 (16.7) | 3 (12.5) | 1 (12.5) | 2 (25.0) | 3 (37.5) | 5 (31.3) | 0 |
| Aspartate aminotransferase increased | 0 | 0 | 2 (33.3) | 2 (33.3) | 4 (16.7) | 0 | 2 (25.0) | 3 (37.5) | 5 (31.3) | 0 |
| White blood cell count increased | 0 | 0 | 2 (33.3) | 2 (33.3) | 4 (16.7) | 0 | 1 (12.5) | 1 (12.5) | 2 (12.5) | 0 |
| Gamma-glutamyl transferase increased | 0 | 0 | 1 (16.7) | 1 (16.7) | 2 (8.3) | 0 | 1 (12.5) | 2 (25.0) | 3 (18.8) | 0 |
| **General disorders and administration site conditions** | **0** | **2 (33.3)** | **4 (66.7)** | **6 (100)** | **12 (50.0)** | **0** | **7 (87.5)** | **8 (100)** | **15 (93.8)** | **0** |
| Pyrexia | 0 | 0 | 2 (33.3) | 6 (100) | 8 (33.3) | 0 | 1 (12.5) | 1 (12.5) | 2 (12.5) | 0 |
| Injection site reaction | 0 | 0 | 3 (50.0) | 1 (16.7) | 4 (16.7) | 0 | 5 (62.5) | 7 (87.5) | 12 (75.0) | 0 |
| Injection site pain | 0 | 2 (33.3) | 0 | 0 | 2 (8.3) | 0 | 3 (37.5) | 2 (25.0) | 5 (31.3) | 0 |
| Injection site erythema | 1 (16.7) | 0 | 1 (16.7) | 0 | 2 (8.3) | 0 | 4 (50.0) | 3 (37.5) | 7 (43.8) | 0 |
| Injection site swelling | 0 | 0 | 0 | 1 (16.7) | 1 (4.2) | 0 | 0 | 2 (25.0) | 2 (12.5) | 0 |

Abbreviations: AE, adverse event; MAD, multiple ascending dose; SAD, single ascending dose; TEAE, treatment-emergent adverse event.

**Supplementary Table 4: Treatment-emergent adverse events reported in ≥2 CHB patients in any treatment cohort by SOC and PT (Phase 1b)**

|  | **Phase 1b – NA-treated HBeAg-negative CHB patients** | | | | | |
| --- | --- | --- | --- | --- | --- | --- |
| **System Organ Class/**  **Preferred Term, n (%)** | **150 mg** | **300 mg** | **300 mg- HighS** | **Pooled 300 mg** | **Total AHB-137** | **Pooled Placebo** |
|  | **N=8** | **N=8** | **N=2** | **N=10** | **N=18** | **N=4** |
| Participants with at least one TEAE | 8 (100) | 8 (100) | 2 (100) | 10 (100) | 18 (100) | 1 (25.0) |
| **General disorders and administration site conditions** | **7 (87.5)** | **7 (87.5)** | **2 (100)** | **9 (90.0)** | **16 (88.9)** | **0** |
| Injection site reaction | 5 (62.5) | 2 (25.0) | 1 (50.0) | 3 (30.0) | 8 (44.4) | 0 |
| Injection site erythema | 2 (25.0) | 4 (50.0) | 1 (50.0) | 5 (50.0) | 7 (38.9) | 0 |
| Pyrexia | 1 (12.5) | 3 (37.5) | 2 (100) | 5 (50.0) | 6 (33.3) | 0 |
| Injection site pain | 4 (50.0) | 2 (25.0) | 0 | 2 (20.0) | 6 (33.3) | 0 |
| **Investigations** | **3 (37.5)** | **7 (87.5)** | **2 (100)** | **9 (90.0)** | **12 (66.7)** | **0** |
| C-reactive protein increased | 2 (25.0) | 6 (75.0) | 2 (100) | 8 (80.0) | 10 (55.6) | 0 |
| Lymphocyte count decreased | 0 | 4 (50.0) | 2 (100) | 6 (60.0) | 6 (33.3) | 0 |
| Alanine aminotransferase increased | 1 (12.5) | 3 (37.5) | 1 (50.0) | 4 (40.0) | 5 (27.8) | 0 |
| Platelet count decreased | 0 | 2 (25.0) | 2 (100) | 4 (40.0) | 4 (22.2) | 0 |
| Aspartate aminotransferase increased | 0 | 2 (25.0) | 1 (50.0) | 3 (30.0) | 3 (16.7) | 0 |
| **Infections and infestations** | **3 (37.5)** | **2 (25.0)** | **0** | **2 (20.0)** | **5 (27.8)** | **1 (25.0)** |
| Upper respiratory tract infection | 3 (37.5) | 2 (25.0) | 0 | 2 (20.0) | 5 (27.8) | 1 (25.0) |
| **Gastrointestinal disorders** | **3 (37.5)** | **0** | **0** | **0** | **3 (16.7)** | **0** |
| Nausea | 3 (37.5) | 0 | 0 | 0 | 3 (16.7) | 0 |
| Vomiting | 2 (25.0) | 0 | 0 | 0 | 2 (11.1) | 0 |

Abbreviations: AE, adverse event; CHB, chronic hepatitis B; TEAE, treatment-emergent adverse event.

**Supplementary Table 5: Treatment-related TEAEs in ≥2 healthy** **volunteers in any treatment cohort by SOC and PT (Phase 1a)**

|  | **Phase 1a - Healthy Volunteers** | | | | | | | | | |
| --- | --- | --- | --- | --- | --- | --- | --- | --- | --- | --- |
| **System Organ Class/**  **Preferred Term, n (%)** | **SAD** | | | | | | **MAD** | | | |
|  | **75 mg** | **150 mg** | **300 mg** | **450 mg** | **Total AHB-137** | **Placebo** | **150 mg** | **300 mg** | **Total AHB-137** | **Placebo** |
|  | **N=6** | **N=6** | **N=6** | **N=6** | **N=24** | **N=8** | **N=8** | **N=8** | **N=16** | **N=4** |
| Participants with at least one TRAE | 0 | 2 (33.3) | 6 (100) | 6 (100) | 14 (58.3) | 1 (12.5) | 7 (87.5) | 8 (100) | 15 (93.8) | 1 (25.0) |
| **Investigations** | 0 | 1 (16.7) | 5 (83.3) | 6 (100) | 12 (50.0) | 1 (12.5) | 4 (50.0) | 7 (87.5) | 11 (68.8) | 1 (25.0) |
| C-reactive protein increased | 0 | 1 (16.7) | 5 (83.3) | 6 (100) | 12 (50.0) | 0 | 4 (50.0) | 5 (62.5) | 9 (56.3) | 1 (25.0) |
| Lymphocyte count decreased | 0 | 0 | 4 (66.7) | 6 (100) | 10 (41.7) | 0 | 2 (25.0) | 4 (50.0) | 6 (37.5) | 0 |
| Neutrophil count increased | 0 | 0 | 2 (33.3) | 4 (66.7) | 6 (25.0) | 0 | 0 | 1 (12.5) | 1 (6.3) | 0 |
| Alanine aminotransferase increased | 0 | 0 | 2 (33.3) | 1 (16.7) | 3 (12.5) | 1 (12.5) | 2 (25.0) | 3 (37.5) | 5 (31.3) | 0 |
| Aspartate aminotransferase increased | 0 | 0 | 2 (33.3) | 2 (33.3) | 4 (16.7) | 0 | 2 (25.0) | 3 (37.5) | 5 (31.3) | 0 |
| White blood cell count increased | 0 | 0 | 2 (33.3) | 2 (33.3) | 4 (16.7) | 0 | 1 (12.5) | 1 (12.5) | 2 (12.5) | 0 |
| Gamma-glutamyl transferase increased | 0 | 0 | 1 (16.7) | 1 (16.7) | 2 (8.3) | 0 | 1 (12.5) | 2 (25.0) | 3 (18.8) | 0 |
| **General disorders and administration site conditions** | 0 | 2 (33.3) | 4 (66.7) | 6 (100) | 12 (50.0) | 0 | 7 (87.5) | 8 (100) | 15 (93.8) | 0 |
| Pyrexia | 0 | 0 | 2 (33.3) | 6 (100) | 8 (33.3) | 0 | 1 (12.5) | 1 (12.5) | 2 (12.5) | 0 |
| Injection site reaction | 0 | 0 | 3 (50.0) | 1 (16.7) | 4 (16.7) | 0 | 5 (62.5) | 7 (87.5) | 12 (75.0) | 0 |
| Injection site pain | 0 | 2 (33.3) | 0 | 0 | 2 (8.3) | 0 | 3 (37.5) | 2 (25.0) | 5 (31.3) | 0 |
| Injection site erythema | 1 (16.7) | 0 | 1 (16.7) | 0 | 2 (8.3) | 0 | 4 (50.0) | 3 (37.5) | 7 (43.8) | 0 |
| Injection site swelling | 0 | 0 | 0 | 1 (16.7) | 1 (4.2) | 0 | 0 | 2 (25.0) | 2 (12.5) | 0 |

Abbreviations: AE, adverse event; MAD, multiple ascending dose; SAD, single ascending dose; TEAE, treatment-emergent adverse event.

**Supplementary Table 6: Treatment-Related TEAEs in ≥2 CHB patients in Any Treatment Cohort by SOC and PT (Phase 1b)**

|  | **Phase 1b - NA-treated HBeAg-negative CHB patients** | | | | | |
| --- | --- | --- | --- | --- | --- | --- |
| **System Organ Class/**  **Preferred Term, n (%)** | **150 mg** | **300 mg** | **300 mg- HighS** | **Pooled 300 mg** | **Total AHB-137** | **Pooled Placebo** |
|  | **N=8** | **N=8** | **N=2** | **N=10** | **N=18** | **N=4** |
| Participants with at least one TRAE | 7 (87.5) | 7 (87.5) | 2 (100) | 9 (90.0) | 16 (88.9) | 0 |
| **General disorders and administration site conditions** | **7 (87.5)** | **7 (87.5)** | **2 (100)** | **9 (90.0)** | **16 (88.9)** | **0** |
| Injection site reaction | 5 (62.5) | 2 (25.0) | 1 (50.0) | 3 (30.0) | 8 (44.4) | 0 |
| Injection site erythema | 2 (25.0) | 4 (50.0) | 1 (50.0) | 5 (50.0) | 7 (38.9) | 0 |
| Pyrexia | 1 (12.5) | 3 (37.5) | 2 (100) | 5 (50.0) | 6 (33.3) | 0 |
| Injection site pain | 4 (50.0) | 2 (25.0) | 0 | 2 (20.0) | 6 (33.3) | 0 |
| **Investigations** | **1 (12.5)** | **6 (75.0)** | **2 (100)** | **8 (80.0)** | **9 (50.0)** | **0** |
| C-reactive protein increased | 1 (12.5) | 5 (62.5) | 2 (100) | 7 (70.0) | 8 (44.4) | 0 |
| Lymphocyte count decreased | 0 | 4 (50.0) | 2 (100) | 6 (60.0) | 6 (33.3) | 0 |
| Platelet count decreased | 0 | 2 (25.0) | 2 (100) | 4 (40.0) | 4 (22.2) | 0 |
| **Gastrointestinal disorders** | **2 (25.0)** | **0** | **0** | **0** | **2 (11.1)** | **0** |
| Vomiting | 2 (25.0) | 0 | 0 | 0 | 2 (11.1) | 0 |
| Nausea | 2 (25.0) | 0 | 0 | 0 | 2 (11.1) | 0 |

Abbreviations: AE, adverse event; CHB, chronic hepatitis B; TEAE, treatment-emergent adverse event.

**Supplementary Table 7: Inject site reaction adverse events in healthy volunteers in Any Treatment Cohort (Phase 1a)**

|  | **Phase 1a - Healthy Volunteers** | | | | | | | | | |
| --- | --- | --- | --- | --- | --- | --- | --- | --- | --- | --- |
| **Preferred Term, n (%)** | **SAD** | | | | | | **MAD** | | | |
|  | **75 mg** | **150 mg** | **300 mg** | **450 mg** | **Total AHB-137** | **Placebo** | **150 mg** | **300 mg** | **Total AHB-137** | **Placebo** |
|  | **N=6** | **N=6** | **N=6** | **N=6** | **N=24** | **N=8** | **N=8** | **N=8** | **N=16** | **N=4** |
| Participants with at least one ISR | 1 (16.7) | 2 (33.3) | 4 (66.7) | 2 (33.3) | 9 (37.5) | 0 | 7 (87.5) | 8 (100) | 15 (93.8) | 0 |
| Injection site reaction | 0 | 0 | 3 (50.0) | 1 (16.7) | 4 (16.7) | 0 | 5 (62.5) | 7 (87.5) | 12 (75.0) | 0 |
| Grade 1 | 0 | 0 | 3 (50.0) | 1 (16.7) | 4 (16.7) | 0 | 0 | 2 (25.0) | 2 (12.5) | 0 |
| Grade 2 | 0 | 0 | 0 | 0 | 0 | 0 | 5 (62.5) | 5 (62.5) | 10 (62.5) | 0 |
| ≥Grade 3 | 0 | 0 | 0 | 0 | 0 | 0 | 0 | 0 | 0 | 0 |
| Injection site pain | 0 | 2 (33.3) | 0 | 0 | 2 (8.3) | 0 | 3 (37.5) | 2 (25.0) | 5 (31.3) | 0 |
| Grade 1 | 0 | 2 (33.3) | 0 | 0 | 2 (8.3) | 0 | 3 (37.5) | 2 (25.0) | 5 (31.3) | 0 |
| Grade 2 | 0 | 0 | 0 | 0 | 0 | 0 | 0 | 0 | 0 | 0 |
| ≥Grade 3 | 0 | 0 | 0 | 0 | 0 | 0 | 0 | 0 | 0 | 0 |
| Injection site erythema | 1 (16.7) | 0 | 1 (16.7) | 0 | 2 (8.3) | 0 | 4 (50.0) | 3 (37.5) | 7 (43.8) | 0 |
| Grade 1 | 1 (16.7) | 0 | 1 (16.7) | 0 | 2 (8.3) | 0 | 4 (50.0) | 3 (37.5) | 7 (43.8) | 0 |
| Grade 2 | 0 | 0 | 0 | 0 | 0 | 0 | 0 | 0 | 0 | 0 |
| ≥Grade 3 | 0 | 0 | 0 | 0 | 0 | 0 | 0 | 0 | 0 | 0 |
| Injection site swelling | 0 | 0 | 0 | 1 (16.7) | 1 (4.2) | 0 | 0 | 2 (25.0) | 2 (12.5) | 0 |
| Grade 1 | 0 | 0 | 0 | 1 (16.7) | 1 (4.2) | 0 | 0 | 0 | 0 | 0 |
| Grade 2 | 0 | 0 | 0 | 0 | 0 | 0 | 0 | 2 (25.0) | 2 (12.5) | 0 |
| ≥Grade 3 | 0 | 0 | 0 | 0 | 0 | 0 | 0 | 0 | 0 | 0 |
| Injection site induration | 0 | 0 | 0 | 0 | 0 | 0 | 0 | 1 (12.5) | 1 (6.3) | 0 |
| Grade 1 | 0 | 0 | 0 | 0 | 0 | 0 | 0 | 0 | 0 | 0 |
| Grade 2 | 0 | 0 | 0 | 0 | 0 | 0 | 0 | 1 (12.5) | 1 (6.3) | 0 |
| ≥Grade 3 | 0 | 0 | 0 | 0 | 0 | 0 | 0 | 0 | 0 | 0 |

Abbreviations: AE, adverse event; ISR, injection-site reaction; MAD, multiple ascending dose; PT, preferred term; SAD, single ascending dose; TEAE, treatment-emergent adverse event.

**Supplementary Table 8: Inject Site Reaction Adverse Events in CHB Patients in Any Treatment Cohort (Phase 1b)**

|  | **Phase 1b - NA-treated HBeAg-negative CHB patients** | | | | | |
| --- | --- | --- | --- | --- | --- | --- |
| **Preferred Term, n (%)** | **150 mg** | **300 mg** | **300 mg- HighS** | **Pooled 300 mg** | **Total AHB-137** | **Pooled Placebo** |
|  | **N=8** | **N=8** | **N=2** | **N=10** | **N=18** | **N=4** |
| Participants with at least one ISR | 7 (87.5) | 6 (75.0) | 1 (50.0) | 7 (70.0) | 14 (77.8) | 0 |
| Injection site reaction | 5 (62.5) | 2 (25.0) | 1 (50.0) | 3 (30.0) | 8 (44.4) | 0 |
| Grade 1 | 4 (50.0) | 1 (12.5) | 0 | 1 (10.0) | 5 (27.8) | 0 |
| Grade 2 | 1 (12.5) | 1 (12.5) | 1 (50.0) | 2 (20.0) | 3 (16.7) | 0 |
| ≥Grade 3 | 0 | 0 | 0 | 0 | 0 | 0 |
| Injection site erythema | 2 (25.0) | 4 (50.0) | 1 (50.0) | 5 (50.0) | 7 (38.9) | 0 |
| Grade 1 | 0 | 2 (25.0) | 1 (50.0) | 3 (30.0) | 3 (16.7) | 0 |
| Grade 2 | 2 (25.0) | 2 (25.0) | 0 | 2 (20.0) | 4 (22.2) | 0 |
| ≥Grade 3 | 0 | 0 | 0 | 0 | 0 | 0 |
| Injection site pain | 4 (50.0) | 2 (25.0) | 0 | 2 (20.0) | 6 (33.3) | 0 |
| Grade 1 | 4 (50.0) | 2 (25.0) | 0 | 2 (20.0) | 6 (33.3) | 0 |
| Grade 2 | 0 | 0 | 0 | 0 | 0 | 0 |
| ≥Grade 3 | 0 | 0 | 0 | 0 | 0 | 0 |
| Injection site swelling | 0 | 1 (12.5) | 0 | 1 (10.0) | 1 (5.6) | 0 |
| Grade 1 | 0 | 0 | 0 | 0 | 0 | 0 |
| Grade 2 | 0 | 1 (12.5) | 0 | 1 (10.0) | 1 (5.6) | 0 |
| ≥Grade 3 | 0 | 0 | 0 | 0 | 0 | 0 |

Abbreviations: AE, adverse event; CHB, chronic hepatitis B; ISR, injection-site reaction; PT, preferred term; TEAE, treatment-emergent adverse event.

**Supplementary Table 9: Analysis of dose proportionality in healthy volunteers (Phase 1a)**

| Dose Range | Parameter (Units) | N | β_0_ | β_1_ Slope | 90% CI of β_1_ |
| --- | --- | --- | --- | --- | --- |
| 75 to 450 mg | AUC_0-inf_ (h*nmol/L) | 23 | 2.7087 | 1.1687 | 1.0332, 1.3042 |
|  | AUC_0-last_ (h*nmol/L) | 24 | 2.7258 | 1.1633 | 1.0312, 1.2954 |
|  | C_max_ (nmol/L) | 24 | 0.6882 | 1.1034 | 0.9463, 1.2604 |

Abbreviations: AUC_0-inf_, area under the plasma concentration–time curve from time zero extrapolated to infinity; AUC_0-last_, area under the plasma concentration–time curve from time zero to the last measurable concentration; C_max_, maximum observed plasma concentration; N, number of participants; PK, pharmacokinetic.

Note: Dose proportionality was assessed using power model with natural log-transformed PK parameters as dependent variables and natural log-transformed dose as the independent variable.

**Supplementary Table 10: Urine PK parameters for healthy volunteers and CHB patients (Pharmacokinetic Parameter Set)**

|  | **Geometric Mean (%CV)^a^** | | | | | |
| --- | --- | --- | --- | --- | --- | --- |
|  | **Ae_0-72h_ (pmol)** | **Ae_0-168h_ (pmol)** | **Fe_0-72h_ (%)** | **Fe_0-168h_ (%)** | **CLr_0-72h_ (L/h)** | **CLr_0-168h_ (L/h)** |
| **Phase 1a - healthy volunteers - single doses** | | | | | | |
| 150 mg (N=6) | 119 (126.6) | NA | 0.569 (126.6) | NA | 0.0217 (119.9) | NA |
| 300 mg (N=6) | 449 (60.5) | 1390 (31.2) | 1.08 (60.5) | 3.35 (31.2) | 0.0367 (61.1) | 0.113 (38.5) |
| **Phase 1a - healthy volunteers - multiple doses** | | | | | | |
| 150 mg Day 1 (N=8) | 119 (61.2) | NA | 0.574 (61.2) | NA | 0.0209 (62.3) | NA |
| 150 mg Day 22 (N=8) | 1130 (26.2) | NA | 5.44 (26.2) | NA | 0.208 (23.9) | NA |
| 300 mg Day 1 (N=8) | 376 (54.2) | NA | 0.904 (54.2) | NA | 0.0340  (53.7) | NA |
| 300 mg Day 22 (N=8) | 2530 (27.2) | 4750 (23.9) | 6.07 (27.2) | 11.4 (23.9) | 0.256 (27.0) | 0.466 (22.9) |
| **Phase 1b - CHB patients - dose escalation** | | | | | | |
| 300 mg Day 1 (N=8) | 550 (69.5) | NA | 1.32 (69.5) | NA | 0.0548 (65.5) | NA |
| 300 mg Day 22 (N=8)^a^ | 3210 (26.2) | 6110 (21.2) | 7.71 (26.2) | 14.7 (21.2) | 0.333 (33.6) | 0.616 (30.8) |

Abbreviations: Ae_0-72h_=total cumulative amount excreted in urine over a specific collection interval 0 to 72 hours post dose; Ae_0-168h_=total cumulative amount excreted in urine over a specific collection interval 0 to 168 hours post dose; CL_r_=Renal clearance; CV=coefficient of variation; Fe_0-72h_=Fraction of the dose of drug excreted in urine from time 0 to time 72 hours; Fe_0-168h_=Fraction of the dose of drug excreted in urine from time 0 to time 168 hours; N=Number of participants; n=Number of participants who had available PK parameters at specified timepoints.

CLr_0-72h_=Ae_0-72h_/plasma AUC_0-72h_; CLr_0-168h_=Ae_0-168h_/plasma AUC_0-168h_.

^a^n=7, as 1 participant in phase 1b did not get complete urine samples at the planned sampling timepoints, thus were not included for analyses.

## Supplementary Figures

**Supplementary Figure 1. Study design.** Note: the Phase 1 component for treatment-naïve CHB patients and the potential higher dose (adaptive) cohorts was not conducted as originally planned (Protocol V3.0); therefore, the study design for this part is omitted. CHB, chronic hepatitis B; HBsAg, hepatitis B surface antigen; MAD, multiple ascending dose; SAD, single ascending dose.

**Supplementary Figure 2. Participant disposition of phase 1a/1b.** ^a^ Phase 1b dose expansion 300 mg cohort was open label where randomization was not applicable, and phase 1a and phase 1b dose escalation were designed as double-blind and randomized. Note: Eligible CHB patients should have baseline HBsAg >100 IU/mL to ≤1000 IU/mL at screening in the phase 1b dose escalation and baseline HBsAg >1000 IU/mL to ≤3000 IU/mL at screening in the phase 1b dose expansion.

**Supplementary Figure 3. Evaluations of selected laboratory parameters (ALT [A], CRP [B], platelet [C],** **neutrophil count [D], and lymphocytes count [E]) overtime during phase 1a - healthy volunteers receiving MAD of AHB-137 (150, 300 mg) or placebo.** ALT, alanine aminotransferase; CHB, chronic hepatitis B; CRP, C-reactive protein.

**Supplementary Figure 4. Mean (±SD) Plasma Concentrations of AHB-137 in (A) healthy volunteers in SAD cohorts (75-450 mg, 0-336 h), (B) healthy volunteers and (C) CHB patients in MAD cohorts (150 and 300 mg, 0-72 h) on days 1 and 18/22.** Error bars represent the SD of the mean; dashed line represents lower limit of quantification.

## Supplementary Methods

### ***Eligibility criteria***

Inclusion and Exclusion Criteria for Healthy Volunteers (Phase 1a)

*Inclusion Criteria:*

1. Participants voluntarily participated in the study, and signed the Informed Consent Form (ICF) prior to screening;
2. Participants were able to comply with all the protocol requirements;
3. Participants (and partners) were willing to take effective contraceptive measures from the screening until at least 6 months after the last dosing;
4. Male or female aged 18-55 when signing ICF;
5. Body Mass Index (BMI) between 18 to 28 kg/m^2^ (inclusive) and body weight equal to or over 50 kg for male and 45 kg for female;
6. Vital signs and physical examination are normal, or abnormal values were not clinically significant.

*Exclusion Criteria:*

1. Smoking history of ≥10 cigarettes per day on average within 3 months prior to screening.
2. Any suspect screening ion of drug component allergy, or allergic constitution (multiple drug and food allergy, and judged by the investigator to be clinically significant) in participants;
3. Any history of illicit drug use, drug abuse, and/or alcohol abuse (defined as consumption of ≥14 units of alcohol per week; 1 unit=285 mL beer, 25 mL spirits, or 100 mL wine);
4. Blood donation or blood loss not less than 400 mL within 12 weeks before screening;
5. Drug administration that changed the activity of liver enzymes within 28 days prior to screening;
6. Use of any prescription drugs, over-the-counter medications, vitamin products, or herbal supplements within 14 days prior to screening;
7. Receipt of any vaccination within 3 months prior to screening or planned vaccination during the study period;
8. Consumption of any special diet affecting drug absorption, distribution, metabolism, or excretion (ADME; e.g., pitaya, mango, grapefruit), or participation in strenuous exercise, or other factors that may influence ADME processes within 2 weeks prior to screening;
9. Participants with major recent changes in diet or exercise habits;
10. Receipt of another investigational drug or device within 3 months before first dosing (interventional treatment);
11. Clinically significant electrocardiogram (ECG) abnormalities on screening ECG;
12. TdP high-risk factors (hypokalemia, hypomagnesemia, decompensated heart failure and acute myocardial infarction), and QTc intervals above 450 msec in participants (judged by investigator based on actual screening conditions);
13. Pregnant (positive pregnancy test), recently ready to conceive, or lactating female;
14. Clinically significant lab examination abnormalities, or other clinically significant diseases discovered within 12 months before screening, including but not limited to gastrointestinal, renal, hepatic, neurological, hematological, endocrinological, tumor, pulmonary, immune, mental, or cardiovascular and cerebrovascular diseases;
15. Positive test at Screening of any of the following: hepatitis B virus surface antigen (HBsAg), hepatitis C virus (HCV antibody or HCcAg), human immunodeficiency virus antigen/antibody, or positive syphilis treponemal antibody with a positive rapid plasma reagin (RPR) or toluidine red unheated serum test (TRUST) (RPR or TRUST performed only if treponemal antibody was positive).
16. Any acute disease or concomitant medication occurred during screening to the first dosing;
17. Consumption of chocolate or any caffeine-containing or xanthine-rich foods or beverages within 24 hours before drug dosing.
18. Alcohol consumption, or positive alcohol test within 24 hours before drug dosing;
19. Positive test for urinalysis (including Morphine, Cannabis) in participants;
20. Other factors resulting in participant becoming unsuitable for the study, determined by the investigator.

Inclusion and Exclusion Criteria for CHB Patients (Phase 1b)

Inclusion Criteria:

1. Participants voluntarily participated in the study, and signed the ICF prior to screening;
2. Participants were able to comply with all the protocol requirements;
3. Participants (and partners) were willing to take effective contraceptive measures from the screening until at least 6 months after the last dosing;
4. Male or female aged 18-65 when signing ICF;
5. Body Mass Index (BMI) between 18 to 32 kg/m^2^ (inclusive) and body weight equal to or over 45 kg for male and 40 kg for female;
6. Documented chronic HBV infection (HBsAg positive) equal to or above 6 months prior to screening, or HBsAg positive and IgM hepatitis B core antibody (HBcAb) negative at Screening;
7. Stable treatment of HBeAg negative CHB patients;
8. Currently receiving single-agent treatment with stable NAs (TDF, TAF, or ETV) for at least 6 months and no changes in the NAs treatment regimen were planned during the trial;
9. Serum ALT≤2×ULN, HBV DNA < 100 IU/mL at screening. Dose increasing stage of Ib: 100 IU/mL <HBsAg≤1000 IU/mL; Dose expansion stage of Ib: 1000 IU/ml < HBsAg≤3000 IU/mL at screening.

Exclusion Criteria:

1. Smoking history of ≥10 cigarettes per day on average within 3 months prior to screening.
2. Any suspect screening ion of drug component allergy, or allergic constitution (multiple drug and food allergy, and judged by the investigator to be clinically significant) in participants;
3. Any history of illicit drug use, drug abuse, and/or alcohol abuse (defined as consumption of ≥14 units of alcohol per week; 1 unit=285 mL beer, 25 mL spirits, or 100 mL wine);
4. Any history of major trauma or major surgery within 3 months prior to screening, or planned surgical procedures during the study period;
5. Blood donation or blood loss not less than 400 mL within 12 weeks before screening; or blood transfusion; or blood donation or blood loss not less than 200 mL within 1 month before screening;
6. Any oligonucleotide or siRNA treatments within 12 months before first dosing;
7. Any immunosuppressing, immunomodulator (e.g., thymosin) or cytotoxic drug administrations within 6 months before first dosing; Vaccination within 1 month prior to screening, or planned vaccination during the study;
8. Receipt of anticoagulant therapy (e.g., Warfarin, Factor Xa Inhibitors or antiplatelet drugs such as Clopidogrel);
9. Any clinically significant liver diseases, including but not limited to hepatitis caused by other pathogenic infections, hemochromatosis, Wilson disease, primary biliary cirrhosis, autoimmune liver diseases, alcoholic liver disease, severe non-alcoholic fatty liver disease, drug-induced liver injury, etc.;
10. Personal history of cirrhosis or progressive hepatic fibrosis (e.g., the participant underwent hepatic histopathological examination, which indicated cirrhosis, or underwent endoscopic examination indicating esophagogastric varices);
11. Confirmation or suspicion of decompensated hepatitis B cirrhosis, including but not limited to hepatic encephalopathy, hepatorenal syndrome, esophageal and gastric variceal bleeding, ascites, primary hepatocellular carcinoma, etc.;
12. History of malignancy within the past 5 years, except for certain tumors that could be cured by surgical resection (e.g., non-melanoma skin cancer, cervical intraepithelial neoplasia, thyroid neoplasm, breast tumor, etc. that were treated without signs of recurrence);
13. Combined sever diseases of circulation, digestion, respiration, urinary, blood, metabolism, immune, nervous system, etc.;
14. Acute infection within 2 weeks prior to screening;
15. Receipt of another investigational drug or device within 1 month before screening (interventional treatment);
16. Laboratorial examination: blood platelet counts <90 x 10^9^/L, absolute neutrophil count <1.3 x 10^9^/L, hemoglobin<90 g/L, serum total bilirubin ≥2 x ULN, albumin<30 g/L, creatinine clearance rate (calculated by MDRD formula) ≤60 mL/min, PT/INR>1.5;
17. Alpha-fetoprotein (AFP) >70 ug/L, or imaging suspicion of malignant hepatic space-occupying;
18. HCV antibody/HCcAg positive, AIDS antigen/antibody positive, or Treponema Pallidum antibody positive and Rapid Plasma Reagin (RPR) or Toluidine Red Unheated Serum Test (TRUST) positive, or Hepatitis D antibody positive;
19. Liver stiffness measurement (LSM) ≥12.4 kPa when screening;
20. Pregnant (positive pregnancy test) or lactating female;
21. Positive test for urinalysis (including Morphine, Cannabis) or alcohol test in participants;
22. Other factors resulted in unsuitable for the study, determined by the investigator.

### ***Sentinel dosing and dose-escalation rules for phase 1a/1b***

Phase 1a SAD

In phase 1a SAD, 4 AHB-137 dose levels (75, 150, 300, 450 mg) were evaluated through the treatment of 4 sequential SAD cohorts (planned N=8 per cohort) where eligible participants in each cohort will be randomly assigned to either receive single AHB-137 or placebo at a ratio of 6:2. In the first cohort, 1 participant receiving AHB-137 and 1 receiving placebo served as a sentinel pair and observed 24 hours before dosing the rest of the cohort (6 participants). Subsequent higher dose cohort may proceed only after all subjects in the preceding cohort have completed the Day 4 tolerability assessment and demonstrated acceptable tolerability.

Phase 1a MAD

Phase 1a had 2 MAD cohorts (N=10, randomization ratio per each cohort as 8:2), initiation of the first MAD cohort was tentatively scheduled once the completion of Day 4 tolerability assessment of the SAD 300 mg cohort. Participants received a total of 6 doses of AHB-137 or placebo with each single dose on Days 1, 4, 8, 11, 15, and 22. The dose on Days 4 and 11 was considered a loading dose, supplementing the 4 weekly doses. Similar to the sentinel rule of SAD cohorts, observation till Day 8 for the first 2 participants without safety concerns was followed by enrollment of the remaining 8 participants. Escalation to the next higher dose occurred only after the preceding cohort had passed the Day 15 tolerability evaluation.

Phase 1b MAD

Same escalation rule with phase 1a MAD cohorts was used for phase 1b dose escalation, which was conducted on the NA-treated HBeAg-negative CHB patients with baseline HBsAg level >100 IU/mL to ≤1000 IU/mL (randomization ratio per each cohort as 8:2). The first MAD 150 mg cohort was initiated after completion of Day 15 tolerability assessment of the SAD 150 mg cohort with a total of 6 doses on Days 1, 4, 8, 11, 15, and 18, follow up was continued till Day 109; after completion of Day 15 tolerability assessment, the MAD 300 mg cohort was initiated as well following the same dosing plan with phase 1a MAD cohorts, follow up was continued till Day 113.

Given that the escalation MAD 300 mg cohort has passed the Day 15 tolerability assessment, phase 1b expansion stage only included 1 MAD 300 mg cohort, where NA-treated HBeAg-negative CHB patients with baseline HBsAg level >1000 IU/mL to ≤3000 IU/mL were enrolled and received open-label AHB-137 300 mg on Days 1, 4, 8, 11, 15, and 22, and followed up by Day 113 for further tolerability and efficacy exploration.

### ***Rationale for Loading Dose Schedule***

The loading dose schedule was based on nonclinical pharmacodynamic and PK/PD evaluations. Following single-dose administration in HBV mouse models, maximal HBsAg reduction was observed at approximately 7 days post-dose, followed by virological rebound, indicating that weekly dosing is required to sustain antiviral activity. Preclinical studies further demonstrated that early repeat dosing, including a Day 4 loading dose, accelerates the kinetics of HBsAg decline and enhances antiviral efficacy. In addition, PK/PD analyses suggest that an additional early loading dose (Day 11) may further facilitate more rapid attainment of effective hepatic drug exposure during the initial treatment phase. Based on these considerations, loading doses on Days 4 and 11 were incorporated to enhance early antiviral response.

### ***Rationale for Dose Selections***

Dose selection for AHB-137 in this phase 1 study was based on an integrated evaluation of preclinical efficacy, pharmacokinetics (PK), and toxicology data, together with established translational approaches for antisense oligonucleotides (ASOs).

In preclinical efficacy studies using HBV mouse models, AHB-137 administered at 20 mg/kg achieved a reduction of approximately >2 log_10_ IU/mL in serum HBsAg. Using established allometric scaling methods for 2’-MOE-modified ASOs (mouse-to-human conversion factor of 5), this efficacious dose corresponds to a human equivalent dose (HED) of approximately 4 mg/kg (approximately 240–280 mg in a 60–70 kg adult). This estimate is consistent with clinical experience with bepirovirsen, where a similar predicted dose (~300 mg) aligned with observed antiviral efficacy, supporting the translational validity of this approach.

Nonclinical toxicology studies further supported dose selection. In a 4-week repeat-dose study in cynomolgus monkeys, the no-observed-adverse-effect level (NOAEL) was 15 mg/kg/week, corresponding to a human equivalent dose of approximately 900–1050 mg. In accordance with regulatory guidance, the starting dose of 75 mg in the single ascending dose (SAD) cohorts was selected to provide a conservative safety margin (<10% of HED).

The dose-escalation scheme in the SAD part (75, 150, 300, and 450 mg) was designed in alignment with prior clinical experience for 2’-MOE ASOs. Based on the favorable tolerability observed in SAD cohorts up to 300 mg, together with the predicted efficacious dose range, 300 mg was selected for further evaluation in multiple-dose cohorts to achieve target exposure associated with antiviral activity. The 150 mg dose was included to characterize the dose–response relationship and to evaluate whether lower exposure levels could provide meaningful antiviral activity with improved tolerability, particularly in the context of repeated dosing.

In phase 1b dose-escalation, 150 mg and 300 mg were selected to further evaluate antiviral activity across the predicted efficacious dose range, following favorable tolerability observed in phase 1a.

### ***PK assessments***

PK assessments included measurement of concentration and PK parameters via plasma and urine samples. For measurement of AHB-137 plasma PK parameters, blood samples was collected in the single ascending dose (SAD) cohorts of phase 1a from day 1 (before dosing and 0.5, 1, 1.5, 2, 3, 4, 6, 8, 12, 14 hours post dosing); and day 2 (24 hours post dosing), day 3 (48 hours post dosing), day 4 (72 hours post dosing); days 8, 15, 22, and 30, or early termination visit. In the multiple ascending dose (MAD) cohorts of phase 1a, blood was collected from days 1 and 22 (before dosing and 0.5, 1, 1.5, 2, 3, 4, 6, 8, 12, 14 hours post dosing); day 2 (24 hours post dosing); day 3 (48 hours post dosing); day 4 (before second dosing); days 8, 11, 15 (before dosing); and day 23 (24 hours post last dosing), day 24 (48 hours post last dosing), day 25 (72 hours post last dosing); days 29, 36, 50, 78 and 113, or early termination visit. For the multiple dose cohorts of phase 1b, blood sampling for the dose-escalation 300 mg cohort and dose-expansion 300 mg cohort followed the same sampling timepoints with the MAD cohorts of phase 1a; in the dose-escalation 150 mg cohort of phase 1b, blood was collected from days 1 and 18 (before dosing and 0.5, 1, 1.5, 2, 3, 4, 6, 8, 12, 14 hours post dosing); day 2 (24 hours post dosing); day 3 (48 hours post dosing); day 4 (before second dosing); days 8, 11, 15 (before dosing); and day 19 (24 hours post last dosing), day 20 (48 hours post last dosing), day 21 (72 hours post last dosing); days 25, 32, 46, 74, and 109, or early termination visit. For measurement of AHB-137 urine PK parameters, urine samples were collected in the phase 1a SAD 150 mg cohort from days 1 to 4, in the SAD 300 mg cohort from days 1 to 8; urine samples were collected in the phase 1a MAD 150 mg cohort from days 1 to 4 and days 22 to 25; in the phase 1a MAD 300 mg cohort and phase 1b MAD 300 mg cohort from days 1 to 8 and days 22 to 29.
